# A Cross-Sectional Study of Circadian Stimulus in Swedish Radiographers’ Light Environment

**DOI:** 10.1177/19375867241278599

**Published:** 2024-09-12

**Authors:** Camilla Krahmer Anes Viseu, Madeleine Selvander

**Affiliations:** 1 Department of Health Sciences, Faculty of Medicine, 5193 Lund University, Lund, Sweden; 2 Department of Clinical Science Malmo, 5193 Lund University, Malmö, Sweden

**Keywords:** circadian stimulus, circadian rhythm, circadian clock, light environment, radiographers

## Abstract

**Background:** Timely light exposure is a vital aspect to achieve better sleep and well-being. As there are risks with a disturbed circadian rhythm and benefits with light settings that stimulate the rhythm, the circadian effective light, circadian stimulus (CS), for radiographers was examined. **Aim:** The aim of the study was to compare radiographers’ light environment on the workstations, at a university hospital in Southern Sweden in the form of CS and relate that to recommendations published by the Swedish Environmental Protection Agency. **Method:** A cross-sectional method has been applied. The measurements for CS were collected in all labs in the radiology department in the middle of January. **Result:** A total of 804 measures were evenly collected resulting in a median for the 19 labs, where the observed median for all labs was 0.091 CS which is significantly lower than the recommended value of 0.3 CS (*p* < .001). Comparing work light settings with maximum light levels in the brightest and darkest labs showed a significant difference (*p* < .001). **Conclusion:** The CS values in the labs, at the radiology department at a university hospital in Southern Sweden, do not reach the recommended values of circadian stimulus published by the Swedish Environmental Protection Agency when the radiographers themselves set the light. There is a potential for improvement as a significant difference could be seen between the chosen level of light and the maximum possible level of light.

## Introduction

Light is the most important factor that influences the body's circadian rhythm (Figueiro et al., 2019). The light we are exposed to through our eyes affects both our sleep and our well-being (Figueiro et al., 2017, 2019; Hadi et al., 2019; LeGates et al., 2014). The radiology department is an area where the light is often adapted and dimmed to create an environment that benefits radiologists’ ability to read images. Adjusting the light also involves adapting and dimming light coming from outside, thus relying to a large extent on the artificial lighting environment (Harisinghani et al., 2004). Not much research has been done on the light in radiology departments, but it is conceivable that there is a certain tradition of dimming the light.

Guidelines from the Commission of European Communities (CECs) suggest lower ambient light levels of around 50 lux, as well as recommendations from the World Health Organization (WHO) advocating for levels around 100 lux when reading diagnostic images (Commission of the European Communities [CEC], 1997; World Health Organization [WHO], 1982). It is important to note that these guidelines are a few decades old, ranging from 25 to 45 years and that there are new technologies that determine other preferred light levels that should be implemented. A subsequent study also indicates that even reduced levels may yield positive outcomes (McEntee et al., 2006).

The radiographer's profession is based on a three-year-long university bachelor education program and the work includes being able to produce correctly portrayed images together with assessing and optimizing the image quality. Beyond this the role of a radiographer in a Swedish context encompasses more than just imaging procedures, they also provide care and support to patients during their time in the X-ray department (Örnberg & Andersson, 2012). As the images are to be assessed in terms of quality, this means that similar lower-intensity lighting conditions for radiographers as for radiologists are probable. Dimmed lighting may also be needed when the radiographer performs the examinations (Söderbäck, 2014).

The human body possesses an internal circadian pacemaker that maintains the body's rhythm and has a cycle of just over 24 hr in most cases (Czeisler et al., 1999; Roenneberg & Merrow, 2016). To maintain the circadian rhythm, it is restored and synchronized by external factors. Light exposure is one of the conditions that is resetting the circadian rhythm (Czeisler et al., 1999; Huang et al., 2011; LeGates et al., 2014; Roenneberg et al., 2003). If the circadian clock is not reset, the circadian rhythm might end up out of sync with the external environment (Duffy & Czeisler, 2009).

The circadian rhythm is closely linked to behavior, sleep, and health (Roenneberg & Merrow, 2016). Important systems in the body are affected by a disturbed circadian rhythm which can lead to negative effects including weight gain, high blood pressure, or diabetes (Evans & Davidson, 2013; Maury, 2019; Potter et al., 2016). The impact of these processes has shown links to increased cancer mortality (Evans & Davidson, 2013; Pariollaud & Lamia, 2020; Roenneberg & Merrow, 2016). Light exposure used as therapy has been shown to reduce fatigue and drowsiness together with a positive effect on depression and better sleep (Duffy & Czeisler, 2009; Figueiro et al., 2017, 2019; Tao et al., 2020).

Circadian stimulus (CS) is a value that connects the effect of light exposure to its biological effect on the suppression of melatonin, which is a sleep/wake-affecting hormone that can be measured in the blood (Acosta et al., 2017; Rea & Figueiro, 2018) and therefor gives a measure of the effectiveness of the retinal light stimulus (Figueiro et al., 2019). The CS values range from 0 to 0.7 and are relative units transformed from CL_A_, the degree to which a light source would suppress melatonin if given for 1 hr at night, is up to 70% (Acosta et al., 2017; Rea & Figueiro, 2018). Different wavelengths of light together with different light intensities have different effects on melatonin suppression (Moore-Ede et al., 2020). The lower wavelengths, around 460 nm, on the spectrum give a higher suppression of melatonin than the higher wavelengths, even though they all influence the suppression (Acosta et al., 2017; Rea et al., 2010). Spectral power distribution describes the combination of intensity and energy at different wavelengths in light (Kusznier, 2018). The CS calculation formula from Lighting-Research-Center (LRC) delivers values for the suppression of melatonin regardless of which light source is used as it utilizes the values for the SPD and photopic illuminance (lux) at the plane of the corneas (Lighting-Research-Center, 2021; Rea & Figueiro, 2018; Rea et al., 2010).

Most people living in an industrialized society, spend the majority of their time indoors (U.S. Environmental Protection Agency, 1989). Indoor light is often inefficient in providing enough entertainment for the circadian rhythm, which can cause circadian disruption (Wright et al., 2013). To strengthen the circadian rhythm light between 8:00 and 12:00 is especially important (Acosta et al., 2017). The Swedish Work Environment Authority has published a knowledge compilation describing the effects of light. In accordance with research on the field, the compilation recommends that the lighting environment provides at least 0.3 CS for at least 1 hr from the beginning of work in the morning until 12:00 at lunch time and does not have an average < 0.1 CS (Lowden, 2019). Similar findings are described in an article by Acosta et al., where they also recommend at least a CS of 0.35 for 1 hr in the morning (Acosta et al., 2017). Both of which are in line with Underwriters Laboratories (UL) guidelines (UL24480) where it's recommended a minimum of 0.3 CS for a duration of at least 2 hr (Underwriters Laboratories, 2019).

Previous articles investigating indoor spaces in educational environments and in-office workers have shown CS levels below the recommended level when there is limited natural daylight (Bellia et al., 2013; Figueiro et al., 2019). The lighting in the radiology department is adapted, dimmed, and relying to a large extent on the artificial lighting environment (Harisinghani et al., 2004; Söderbäck, 2014). At the same time, there are, at the point of writing, no studies that have examined how the lighting and CS conditions are for radiographers.

### Aim

The aim of the study is to compare radiographers’ light environment on the workstations, at a hospital in Southern Sweden, in the form of Circadian Stimulus, relative to the recommendations published by the Swedish Environmental Protection Agency.

### Research Questions

Does CS reach a recommended level when the radiographers themselves set the light?

Does CS reach the recommended level when the light in the work area is set to the highest possible level at the brightest and darkest labs?

Is there a difference in CS between the work light setting and the possible maximum lighting, in the brightest and darkest labs?

## Method

A cross-sectional design with quantitative data has been applied.

The measurements for CS were made in all labs, that were managed by or that radiographers worked in, in the radiology departments at a hospital in Southern Sweden. The rooms have dimmer switches to control the lighting. The lights will turn on at the last setting used and can be adjusted as needed. One examination room together with the control room was considered a lab. Depending on the modality and room, either the control room and the examination room are divided by a door, or it is open to the wall with leaded glass, behind which the staff work. The rooms are separated by leaded windows and the view is mostly over the examination room. In the examination room, the radiographer welcomes the patient, often preparing and positioning as necessary based on the specific examination being conducted. In the control room, the referral is reviewed, the examination is planned, information is documented, and image processing takes place and is completed. The staff turn their gaze both towards the screens they are utilizing for work and around the entire examination room. What proportion of the time is spent in the control vs examination room depends on modality and examination. The staff's regular working hours are from 7:00 a.m. to 4:30 p.m. Depending on the department they work in and their role, it may include one to three shifts a week that are until 20:00 in the evening. The department responsible for emergency operations operates 24 hr a day. Depending on the department they work in and their role, it may include one to three shifts a week that are until 20:00 in the evening. The department responsible for emergency operations operates 24 hr a day, whereupon some radiographers work at night. The modalities of the labs were: conventional X-ray, fluoroscopy, computed tomography (CT), and interventional angiography. The magnetic resonance imaging (MRI) was excluded for safety reasons, due to the strong magnetism, metal components of the measuring equipment, and the absence of the possibility to turn off the machine. The coffee rooms where radiography takes its breaks are not included in the measurements. The radiographers have a shorter break during the morning, which is taken if possible and amounts to 15 min.

The data were collected in the middle of January 2022.

All labs, that were operational and measurable, were included, with the measuring points that were appropriate for the method.

The measurements were done when there was time between patients, without changing the light from the work setting they previously used or set to the highest possible level. There were four out of 19 labs that had windows. These rooms were measured once in sunny weather and once in cloudy weather. The measurements on the labs with windows were measured between 11:00 and 12:00 to extract the maximum possible CS value before 12:00, for these conditions.

**Figure 1.**
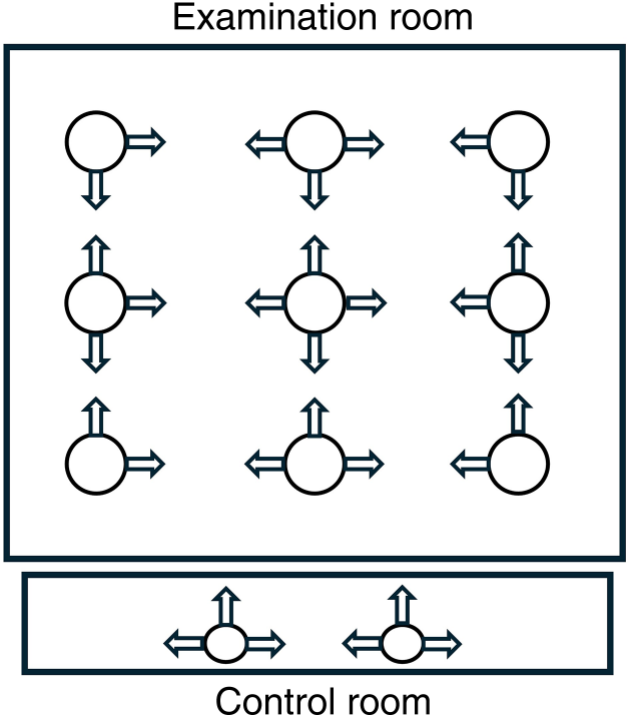
Measuring points and angles in the examination and control room.

The data were measured in lux and SPD for each measuring point and angle, for control rooms at the two heights, and for the examination rooms separately. These measuring points are illustrated in Figure 1. The method of how to conduct the measurements was based on a combination of two methods used in two separate articles (Acosta et al., 2017; Zeng et al., 2021).

The data collected were measured with a spectroradiometer (XL-500H BLE Spectroradiometer, nanoλ, South Korea). The spectral power distribution was measured with wavelengths ranging from 390 to 760 nm. The illuminance level was measured in lux. This device is a spectroradiometer that is wirelessly linked to a mobile application through Bluetooth technology (nanoλ, 2024). The spectroradiometer was placed on a camera stand equipped with a spirit level to ensure accurate alignment at consistent heights. This allowed for standardized measurements to be taken at the same height and angle for each reading.

The measurements of lux and SPD were converted to the outcome CS, according to the model by Rea and Figueiro, using the Lighting-Research-Center calculator for CS (Lighting-Research-Center, 2021; Rea & Figueiro, 2018; Rea et al., 2010).

Measurements were done in both examination and in control rooms separately (see Figure 1). Measurements in the control rooms were made vertically at two points, at a height of 1.2 m, in three directions. This was to get an estimate of the general lighting seen during sedentary work. In the same way, measurements were also done at a height of 1.6 m, to get an estimate of the work performed standing up. These measurements capture the areas of focus for radiographers.

In the examination room, the corresponding measurements were made in the center of the room in four directions vertically at a height of 1.6 m. In addition, four corner points, at the same height, 1 m out from the walls, were measured in two directions. Between these corner points, there were measurements made in three directions. A total of nine measuring points for the room were made.

**Figure 2.**
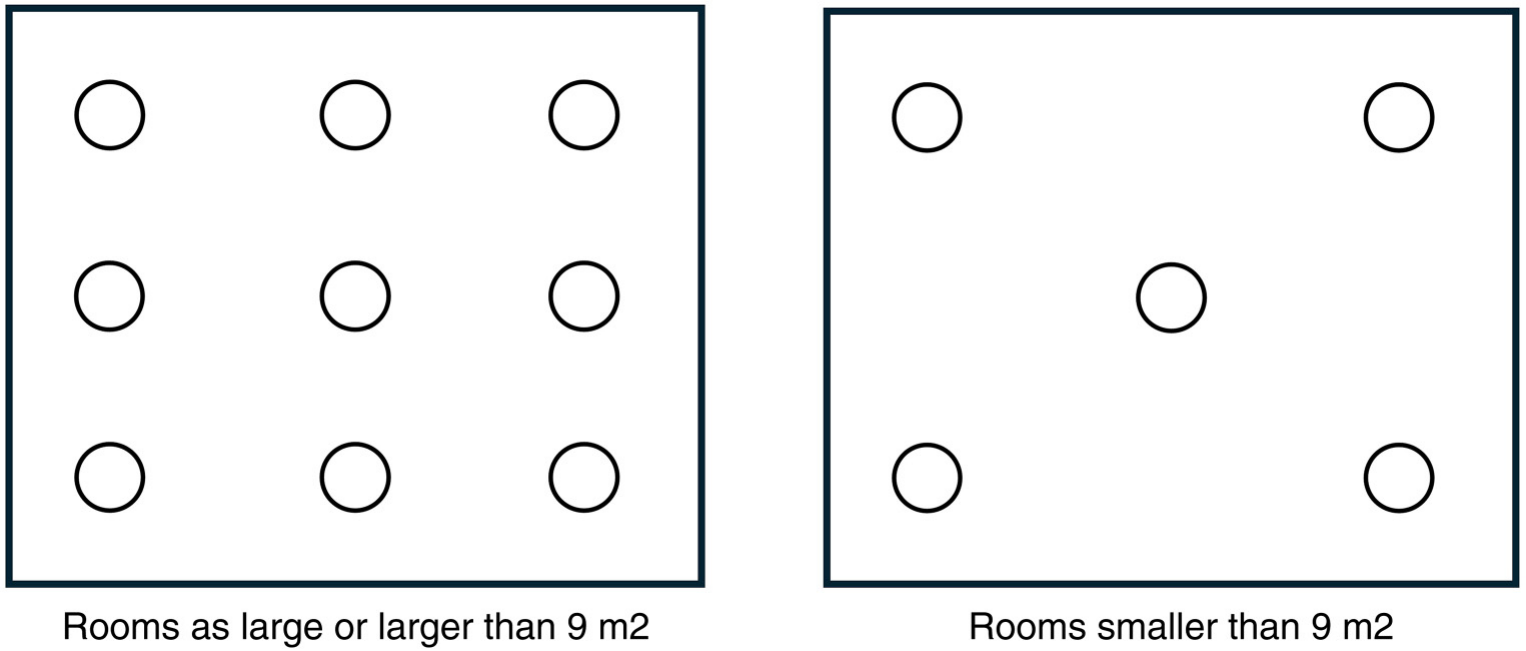
Measurement points for general measurements of illuminance.

To find out which rooms appeared more on the darker and brighter side, general measurements of the lighting in the rooms were done. These measurements were used to compare the general value of the illuminance level in the measure of lux for the examination and control room combined, at each lab. To get the general values, the rooms that were equal to or over 9 m^2^, were calculated at nine points. Which was all the examination rooms and six of the control rooms. Those that were smaller than this area were calculated with five measuring points. Only 13 of the control rooms were under 9 m^2^, of all rooms measured. The measuring points are shown in Figure 2. These general measurements were made horizontally at a height of 1 m, to follow the standard and were made on the work light setting of the light.

The values for these points were added together for the control room and examination room separately, then they were divided by the number of measured points. After this, the values for the control room and examination room are added together to be divided into two. This gave general lighting for the room, without any weight being attached to one of the rooms, due to the possible uneven number of measuring points.

Analysis was made in Statistical Package for the Social Sciences (SPSS) 27. The analysis was made of values in CS. A confidence interval of 95% was applied.

Comparisons against recommended CS values were done with a one-sample Wilcoxon sign rank test. The measurements made were compared against the already known recommended value of 0.3 CS.

Light set to the highest level and the usual work light are described using descriptive statistics, this to show the possible light range. A one-sample Wilcoxon sign rank test was used to see if the light in the brightest or darkest labs reached the recommended value in CS.

The difference in CS, between the work light setting and the maximum possible level of lighting, in the two examined labs has been analyzed using the non-parametric Mann-Whitney *U* test (Björk, 2020; Mackridge & Rowe, 2018).

Ethical approval was not necessary as the study did not involve any research persons.

## Results

The number of points measured was 804, of these only 67 of the measures were above the recommended value and all of the median values for the labs were below the recommended value of 0.3 CS (Table 1).

**Table 1.** Median Calculated by One-Sample Wilcoxon Sign Rank Test.

Modality	*N*	Median	*p*
CT control room	120	0.139	<.001
CT examination room	216	0.151	<.001
Conventional control room	84	0.054	<.001
Conventional examination room	168	0.077	<.001
Fluoroscopy control room	24	0.022	<.001
Fluoroscopy examination room	48	0.029	<.001
Intervention/angiography control room	48	0.064	<.001
Intervention/angiography examination room	96	0.105	<.001

Abbreviation: CT = computed tomography.

All 19 labs were also analyzed to determine whether the CS median for the work light setting was different from the recommended value of 0.3 CS. The observed median was 0.091 CS with a range of 0.628. This is significantly lower than the recommended value of 0.3 CS (*p* < .001). This suggests that the set lighting at the labs for radiographers is below the recommended value in CS.

Based on the analysis of the measurements on the general illuminance level, illuminance measured in lux (*M* = 164, *SD* = 128), it was concluded that the lab with the lowest level of the illuminance measured 46 lux at a general and the lab with the highest level of illuminance measured 510 lux at a general.

The CS values for both the lab that appeared more on the darker side and the lab that appeared more on the brighter side with the work light setting were significantly lower than the recommended value with a median of 0.04 (range = 0.025; *p* = .003) for the darker lab and a median of 0.119 (range = 0.117; *p* = .003) for the brighter lab.

The median CS for the lab that appeared more on the brighter side with the highest illuminance level was 0.296 CS with a range of 0.234. This is not significantly lower than the recommended value (*p* = .108). For the darkest lab with the highest illuminance level, the corresponding median value for CS was 0.106 with a range of 0.088, this is significantly lower than the recommended value (*p* < .003). None of the work light settings at the labs reaches the recommended CS level. Only around one-third of the measurements for the brightest room with the highest level of illuminance gave values above the recommended level of 0.3 CS.

*None of the work light settings at the labs reaches the recommended CS level. Only around one-third of the measurements for the brightest room with the highest level of illuminance gave values above the recommended level of 0.3 CS*

Comparing the work light setting with a maximum light level in the lab that appeared more on the bright side showed a significant difference (*p* < .001), for the 11 points measured at each setting, with the light on the highest possible setting and at work light setting. In the same way, there was a significant difference between the two settings for the lab that appeared more on the darker side (*p* < .001). The analysis also indicates that at work light setting, none of the measurements reaches the recommended value of 0.3 CS.

## Discussion

We have investigated CS levels in a normal working environment for radiographers and found that they are lower than the recommended levels published by the Swedish Environmental Protection Agency. At the work light setting, none of the measured labs reached the recommended level of 0.3 CS. The same applied for the darkest lab also when the light setting was set to maximum. Furthermore, we found a significant difference between the work light setting and the maximum possible lighting available.

*We have investigated CS levels in a normal working environment for radiographers and found that they are lower than the recommended levels published by the Swedish Environmental Protection Agency*

As the CS does not reach the recommended level in the lab where the radiographers work, it may possibly have a negative effect on both their sleep and well-being (Figueiro et al., 2017, 2019; Hadi et al., 2019; LeGates et al., 2014; Potter et al., 2016). An improved light environment may therefore have a positive effect on their well-being (Daut & Fonken, 2019; Figueiro et al., 2017, 2019; Raj, 2006; Tao et al., 2020).

However, this is something that needs to be investigated further as different forms and amounts of light can affect, for example, sleep and mood. It is important to consider that not only morning light can influence the circadian rhythm, but also the duration and intensity of light exposure can affect this rhythm (Figueiro et al., 2017).

Similarly, elevated levels of LED lighting have been found to have a negative effect on sleepiness and circadian timing (Chang et al., 2015).

Therefore, it is also necessary to assess how the staff may be impacted by these CS levels, in accordance with the published recommendations, before making any conclusions on this matter.

In the same way focuses this study solely on comparing how the CS levels the X-ray nurses worked under were in the X-ray departments that were investigated and then in relation to the recommendations published by the Swedish Work Environment Authority.

It is important to note that this study is limited in its scope, as it does not allow for direct comparisons or conclusions to be drawn regarding CS levels in other departments or settings when it comes to, for example, sleepiness level. One potential avenue for future research could involve utilizing surveys or assessment forms to evaluate employee well-being, including factors such as sleepiness levels among staff members, together with various light recipes. This method allows for an evaluation of employees’ perceptions of their own levels of sleepiness related to their light environment, and the registered CS values and enables comparisons with findings from other relevant studies.

According to Amarpreet S. Chawla, the ideal level of illuminance is between 75 and 150 lux for radiologists when reading images (Chawla & Samei, 2007). Based on the same conditions for radiographers as for radiologists and comparing with the illuminance levels found in our labs, theoretically, there is a possibility to increase the set lighting in 11 of the 19 examined labs, if we consider their calculations on the horizontal planes. Nevertheless, in line with the guidelines established by both the CEC and the WHO, which recommend levels between 50 and 100 lux, the measured values are deemed plausible and reasonable at least for radiologists (CEC, 1997; WHO, 1982).

*Theoretically, there is a possibility to increase the set lighting in 11 of the 19 examined labs*

As a difference can be seen between the light the radiographers choose to work in and the maximum possible lighting available, there may be room for higher levels of illuminance and thus higher CS values, in relation to the light in which it is chosen to work. Even though most of the measurements for the lab that appeared more on the darker and brighter side did not reach the recommended values in CS at maximum illumination intensity, this study shows that it may be possible to increase CS value in some of the labs if the light, in which the radiographers chose to work in, is set to the highest level.

*As a difference can be seen between the light the radiographers choose to work in and the maximum possible lighting available, there may be room for higher levels of illuminance and thus higher CS values, in relation to the light in which it is chosen to work*

As the study shows that the radiographers do not choose to work under the highest light intensities possible, there are probably reasons for this. The readability of images is one (Harisinghani et al., 2004; Örnberg & Andersson, 2012; Söderbäck, 2014). What other reasons might be, should be investigated. Also, the perspective of the patient being in a vulnerable position, where a lot of light might not be desired, needs to be considered when deciding on light settings. Further studies are required to investigate the reasons for the chosen work light setting before an assessment of the measure can be made.

One possible solution to the lighting might come from a small study that was carried out in a similar environment in the field of radiotherapy, which showed perceived benefits for the health and well-being for staff as they received light therapy using light glasses for a maximum of 30 min between 7 and 9 o'clock (Bragard & Coucke, 2013). A similar technique could later be evaluated as a possible solution, depending on what is found as the reason for the selected dimmed work light.

An important aspect to consider when implementing changes in the lighting environment is how staff are affected and what the consequences may be for them. Further studies are therefore required to investigate the reasons for the chosen level of the setting before an assessment of the measure can be made.

The present study has been carried out in one hospital and although many different modalities, departments, and preferences have been included, other conditions may exist in other hospitals and thus limit the results to indoor environments with similar lighting conditions. However, this study relates to similar findings from a study in an office environment where that study showed values below 0.3 CS in the overcast sky and indoor lighting (Zeng et al., 2021). Similarly, a second study, done in an educational environment, showed light levels below 0.3 CS when examining electric lighting alone, whereas combined electrical and window input gave higher values (Bellia et al., 2013). A strength against the two above-mentioned studies is the number of surfaces that have been measured for this study. As the other two studies have concentrated on certain specific positions and made more in-depth measurements there, this study provides a better picture of the entire work surface in detail. However, possible variations over the day are missed instead, but as this is not relevant for this study and windows are very limited, this is not considered a problem.

As there is a lack of existing research on the light in radiology departments in Sweden, it is not possible to make comparisons with other similar departments. Further studies are still needed at different hospitals for an in-depth understanding of CS for radiographers.

Additionally, measurements were solely conducted within the specific departments of the hospital where our study was conducted, as per the established protocol. Conducting further studies on lighting in other departments of the hospital could provide valuable insights for comparison and analysis.

The strength of this study is that it includes all labs excluding MRI, in the hospital that were running at the time of measurement. This includes a wide range of different light settings and preferences. This could also, conversely, be seen as a limitation, depending on which person is in the lab, the preferences for the settings of the light also change and this can thus affect differences in the values. In the same way, there is probably a difference in CS for the four rooms with windows with regard to seasonal variations. Measurements in this study were made during the darker part of the year. A supplementary study during the lighter part of the year could be a possible next step to get an overall picture for the year.

## Conclusions

The CS in the labs at the radiology department at a hospital in Southern Sweden does not reach the recommended values published by the Swedish Environmental Protection Agency when the radiographers themselves set the level of the light. Neither did the labs that appeared more on the darker and brighter side, with the light at maximum level, reach the recommended CS level. Interestingly, the personnel in all departments/labs, which was investigated in this study, opted not to use the light at its maximum setting and therefore did not choose to work under the highest possible illuminance level available to them. Therefore, this study indicates that the radiographers choose to work in a lighting environment with lower CS than could be available. Further studies are needed to investigate what makes radiographers choose this lighting, in order to further explore the possibilities for improvement.

As there is limited previous knowledge about the light environment for radiographers, this adds important findings regarding the work environment that might influence health-related issues and needs to be investigated further. One could presume that this might not only be a regional problem but could be of interest for radiographers in Europe and the rest of the world.

To our knowledge, this is the first time this type of study, examining the light environment, has been done in the radiology department, for radiographers. This is an unexplored area, where different angles would promote a continued development in the health and well-being of radiographers.

## Implications for Practice

- Since it is shown that there are lower light levels than recommended, it is also important to examine the light environment in other X-ray departments in other locations, as well as in other departments within the healthcare system.
- This study identified an environment that would be possible to use to further study the impact of light on well-being.
- This study shows the importance of further investigating the reasons why radiographers choose a lower light level.
